# Optimized Protocol for Microalgae DNA Staining with SYTO9/SYBR Green I, Based on Flow Cytometry and RSM Methodology: Experimental Design, Impacts and Validation

**DOI:** 10.3390/mps5050076

**Published:** 2022-09-27

**Authors:** Yob Ihadjadene, Thomas Walther, Felix Krujatz

**Affiliations:** 1 Institute of Natural Materials Technology, Technische Universität Dresden, 01069 Dresden, Germany; 2 Biotopa gGmbH—Center for Applied Aquaculture & Bioeconomy, 01454 Radeberg, Germany; 3 Faculty of Natural and Environmental Sciences, University of Applied Sciences Zittau/Görlitz, 02763 Zittau, Germany

**Keywords:** algae, *Chromochloris zofingiensis*, flow cytometry, DNA staining, SYBR Green, SYTO9

## Abstract

Multiple fluorochromes are extensively used to investigate different microalgal aspects, such as viability and physiology. Some of them can be used to stain nucleic acids (DNA). Well-known examples are SYBR Green I and SYTO 9, the latter of which offers several advantages, especially when combined with flow cytometry (FCM)—a powerful method for studying microalgal population heterogeneity and analyzing their cell cycles. However, the effects of these dyes on the microalgae cell physiology have not been fully elucidated yet. A statistical experimental design, using response surface methodology (RSM) with FCM was applied in this study to optimize the DNA staining of a non-conventional microalgae, *Chromochloris zofingiensis,* with SYBR Green I and SYTO 9, and to optimize the variables affecting staining efficiency, i.e., the dye concentration, incubation time and staining temperature. We found that none of these factors affects the staining efficiency, which was not less than 99.65%. However, for both dyes, the dye concentration was shown to be the most significant factor causing cell damage (*p*-values: 0.0003; <0.0001) for SYBR Green I and SYTO 9, respectively. The staining temperature was only significant for SYTO 9 (*p*-value: 0.0082), and no significant effect was observed regarding the incubation time for both dyes. The values of the optimized parameters (0.5 µM, 05 min and 25 °C) for SYTO 9 and (0.5 X, 5 min and 25 °C) for SYBR Green I resulted in the maximum staining efficiency (99.8%; 99.6%), and the minimum damaging effects (12.86%; 13.75%) for SYTO 9 and SYBR Green I, respectively. These results offer new perspectives for improving the use of DNA staining fluorochromes and provides insights into their possible side effects on microalgae.

## 1. Introduction

Microalgae are photosynthetic microorganisms that constitute the basis of life in a variety of marine and freshwater ecosystems [1,2]. They are becoming increasingly important in the bioeconomy and biotechnology sector as an attractive, sustainable source of value-added products [1,2,3,4], owing to their enormous potential for the production of industrially relevant, high-value products, e.g., pigments with antioxidant and antibacterial activity such as carotenoids (astaxanthin, canthaxanthin, β-carotene and lutein) [5,6,7], polysaccharides (hydro colloids, e.g., sulfated polysaccharides) [8] and polyunsaturated and omega-3 fatty acids (e.g., eicosapentaenoic acid or docosahexaenoic acid) [9,10]. Thus, to develop feasible algae-based bioprocesses, it is crucial to study their intrinsic characteristics, such as their physiology, metabolism and their response to factors influencing their growth [4,11,12,13]. Therefore, the analysis and examination of these different key parameters must be accurate and fast [4,14]. One already well-established method for analyzing and examining different physiological state parameters—such as the cell size, granularity and the viability of various microorganisms, including microalgae—is flow cytometry (FCM) [4,11,12,13]. The potential of this technology is important, as it allows high-throughput analysis and the acquisition of key data on cell populations at a single-cell level, based on optical properties such as forward and side scattered light, which serves as an indicator of the cell size distribution and cell granularity [1,13,14,15,16,17,18]. By applying fluorescent markers, cell properties such as viability, storage compounds and DNA content can be determined [4,15,16,17,19,20,21,22]. A wide variety of fluorescent dyes are available for FCM applications [4,16,23,24,25]. However, it is important to understand their operating mode and their interaction with both the environment and the cells in order to obtain accurate staining results, especially since their effects on the physiology of microalgae cells have yet to be fully elucidated [15,16,26]. For this purpose, critical factors to be considered for the selection of an appropriate dye are the cells’ stability and their sensitivity to the staining conditions, no matter what microorganism is being studied [4,15,16,27]. When staining microalgae for cytometric analysis, the ideal fluorochrome must show a high sensitivity towards the target cell parameter of interest, be non-toxic and have maximal fluorescence emission intensities outside of the absorption maximum of the pigments, to avoid spectral interferences [4,25,28,29].

Microalgal nuclear DNA has become an important cell parameter to study for a number of reasons, including when selecting strains with potentially higher secondary metabolite production [30], cell cycle determination [30,31,32,33,34] and in cell culture research to develop feasible algae-based bioprocesses [6]. Flow cytometry is one of the most suitable methods for precisely and rapidly estimating the nuclear DNA content of whole cells, by staining the DNA with a fluorochrome that binds to it [18,28,30,31,32,33,35]. Many DNA-binding dyes can be used to stain nucleic acids. Well-known examples are SYBR Green I (Ex 497 nm/Em 520 nm), DAPI (Ex 358 nm/Em 461 nm), PicoGreen (Ex 480 nm/Em 520 nm) and SYTO 9 (Ex 483 nm/Em 503 nm), which are extensively used due to their good membrane permeability and their compatibility with almost all bench-top flow cytometers [4,12,15,16,17,36,37,38,39]. However, a review of the current literature revealed a wide range of discrepancies in staining methods, with varying results and limited comparability [21,22,23,26,27,39,40,41,42,43,44,45,46,47,48,49,50], highlighting the need for standardized, reproducible protocols. Moreover, only a small number of protocols give an explanation of why staining parameters such as dye concentration, staining time and temperature were chosen, or how stable the added fluorochromes were [16].

Therefore, one possible solution is to employ certain mathematical and statistical approaches that provide more accurate results, such as response surface methodology (RSM) [51,52,53,54]. This approach is widely used in current research and process development in several disciplines, such as environmental biotechnology [55], waste water treatment and bioprocess engineering [54,56]. It is used to model and analyze problems in which the optimal response is affected by several different independent variables [56,57,58], by performing a set of experiments to find the best levels of variables and achieve optimal conditions [59].

With respect to the above-mentioned issues, a statistical experimental design, using RSM methodology and FCM analyses, was applied in this study to optimize the DNA staining of a non-conventional microalgae, *Chromochloris zofingiensis,* with SYBR Green I and SYTO 9, and to optimize the variables affecting staining efficiency, i.e., the dye concentration, staining time and temperature, with the aim of providing a standardized, quick, accurate and reproducible protocol.

## 2. Experimental Design

Examining the literature, we noticed that RSM had not been used to establish and validate staining methods applied to microalgae, or to predict how the staining conditions and staining affect the cell physiology. Applying a wide range of staining dye concentrations, which were applied in the literature, we observed that the dyes could have a negative effect on microalgae cells at certain concentrations (data not shown). For this reason, to maximize the staining efficiency and minimize the damaging effect of the dyes, a central composite design (CCD), based on RSM, was employed to optimize the input factors, as follows: the dye concentration, incubation time and staining temperature. Experimental runs with six replications at the center points were designed for these factors. Specific values were assigned to their ranges based on data from the literature and observations from our preliminary experiments (Table 1). Design-Expert software (Version 13) was employed to code the CCD.

### 2.1. Materials and Reagents

- *Chromochloris zofingiensis* SAG 211-14 (SAG culture strains collection, Göttingen, Lower Saxony, Germany).
- Dimethyl sulfoxide (DMSO) ROTIPURAN^®^ ≥ 99.8% (Carl Roth GmbH + Co. KG, Karlsruhe, Baden-Württemberg, Germany, article number 4720.1).
- Bristol’s Modified (BM) medium (see [60] for the detailed composition).
- 0.9% NaCl solution.
- SYBR^TM^ Green I Nucleic Acid Gel Stain (Thermo Fisher Scientific, Waltham, MA, USA; Cat.no.: S7563).
- SYTO^TM^ 9 Green Fluorescent Nucleic Acid Stain (Thermo Fisher Scientific, Waltham, MA, USA; Cat.no.: S34854).

### 2.2. Equipment

- CyFlow Cube 8, equipped with 488 nm solid laser (SYSMEX GmbH, Norderstedt, Schleswig-Holstein, Germany).
- Eppendorf Thermomixer Comfort (Eppendorf AG, Hamburg, Germany; Cat.no.: 5355).
- GENESYS^TM^ 150 UV-Visible Spectrophotometer (Thermo Fisher Scientific, Waltham, MA, USA; Cat.no.: 840-300000).

## 3. Procedure

### 3.1. Strain Cultivation Conditions

The batch cultivation of *Chromochloris zofingiensis* SAG 211-14 was performed in a 100 mL sterile Erlenmeyer flask, using 50 mL BM medium (composition described by [60]), in a 16 h day/8 h night cycle, 50 µmol photons m^−2^ s^−1^ photon flux density and a temperature of 25 °C, continuously mixing the culture at 150 rpm.

### 3.2. Preparation of Stock Solutions

1. The stock solutions of SYBR Green I (100 X) and Syto 9 (10 µM) were prepared in DMSO in opaque plastic Falcon tubes, from an initial commercial solution of (10,000 X) and (5 mM), respectively. They were transferred to 1.5 mL, opaque Eppendorf tubes and frozen at −20 °C, for storage.

⚠ **CRITICAL STEP:** it is preferable to avoid the use of glass materials during preparation to prevent dye adsorption on the walls.
⚠ **CRITICAL STEP:** both dyes should be protected from light when preparing, using and storing the stock solutions to avoid their photodegradation.

### 3.3. Sample Preparation

2. Cell broth from the shake flasks was diluted in a 0.9% NaCl solution, in a plastic Falcon tube, to a cell density of 7.5 ∗ 10^6^–8 ∗ 10^6^ cells/mL (OD_750nm_ approx. 0.03.)

### 3.4. Sample Staining

3. To stain 2 mL of sample solution, we calculated the volume of dye stock solution to be used, corresponding to the final concentration for every run, according to the CCD design (Table 2).

⚠ **CRITICAL STEP:** before use, allow the tubes containing the stock solution to warm at room temperature and then briefly centrifuge before every use to mix the DMSO with the dye.

4. After staining, put the Eppendorf tubes in the Thermomixer, at 350 rpm, for continuous mixing, with an incubation time and staining temperature according to the CCD design (Table 2).

⚠ **CRITICAL STEP:** at the end of the incubation time and before cytometric measurement, centrifuge briefly to obtain a homogeneous mixture.

### 3.5. Cytometric Measurements

5. Chlorophyll fluorescence was measured in the FL3 channel (675 ± 50 nm) and dye fluorescence was measured in the FL1 channel (520 ± 20 nm), as were forward scattered (FSC) and side scattered (SSC) light.

⚠ **CRITICAL STEP:** before starting the measurements, set the flow rate of the cytometer to approximately 10^3^–2 ∗ 10^3^ events per second, to be sure to detect all the cells and avoid detecting duplicates.

### 3.6. Cytometric Data Analyses

**Figure 1:**
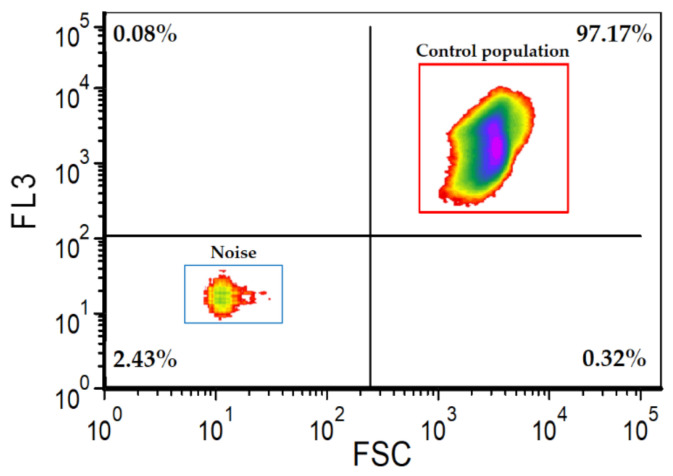
FSC vs. FL3 dotplot with rectangular gates for quantification of microalgae control population.

**Figure 2:**
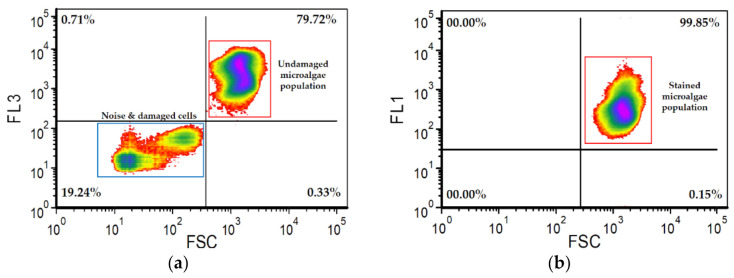
(**a**) FSC vs. FL3 dotplot with rectangular gates for quantification of stained microalgae population. (**b**) FSC vs. FL1 dotplot with rectangular gate for quantification of stained microalgae population only.

6. For each detected event, numerical values for its chlorophyll fluorescence (FL3), dye fluorescence (FL1) and forward (FSC) and side (SSC) scattering were recorded in (fcs) files. FCS Express^TM^ software (De Novo Software, Pasadena, CA, USA) was used to process these collected data.
7. The microalgae population was gated in the two-dimensional FSC vs. FL3 dotplot to remove the background noise (Figure 1), and stained cells were gated in the FSC vs. FL1 dotplot, considering only the microalgae population area (Figure 2b).

⚠ **CRITICAL STEP:** cytometric analysis of at least a triplicate of the microalgae sample without staining is required in order to estimate the microalgae population that will serve as a control. In this study, the microalgae control population represented 97% ± 0.3 (values were calculated based on a triplicate).

#### 3.6.1. Damaged Cells

8. The number of damaged cells was determined after staining by deducting the percentage of the undamaged microalgae population of each sample (Figure 2a) from that of the control population (Figure 1).

#### 3.6.2. Staining Efficiency

9. The staining efficiency was determined after staining by estimating what percentage of the undamaged microalgae population have cells with stained DNA (Figure 2b).

### 3.7. Statistical Analysis

The statistical effect of each factor was determined with the analysis of variance (ANOVA) method, using Design-Expert software (Version 13), at a confidence coefficient level of α = 0.05. The model validity (lack of fit) and the explained variation (R^2^) were used to assess the accuracy of the model fit. To confirm the quality of the validated protocols, experiments were conducted under the predicted optimal conditions, and compared to the predicted model outcomes.

## 4. Results and Discussion

The experimental basis for the RSM approach was provided by the 40 runs carried out while varying the following input variables: the dye concentration, the incubation time and the staining temperature. The results of statistical analysis, as well as the modeling and the validation of the model predictions for the final protocols of both dyes, are presented in the following sections.

### 4.1. DOE—Output Responses and Model Fitting

The experimental results obtained are presented in Table 2. They showed a huge variance in the number of damaged cells, depending on the staining conditions applied, in contrast to the staining efficiency, which exhibited small variations. The percentage of damaged cells ranged from 14.16% to 70.52% and from 9.6% to 32.71% for SYBR Green I and SYTO9, respectively. Meanwhile, the staining efficiency ranged from 99.65% to 99.87% and from 99.71% to 99.87% for SYBR Green I and SYTO9, respectively.

The experimental data were used to identify model equations for the damaging effect on microalgae cells only, as the staining efficiency was satisfactory (not less than 99.65%). These data were fitted to different models, including linear, two-factor interaction (2FI), quadratic and cubic models. The model summary statistics and the lack-of-fit tests for these models are presented in Table 3. For the lack-of-fit test, the *p* > 0.05 indicates that the model is significant, at a 95% confidence interval. The linear model was fitted for both dyes, yielding an estimated R^2^ of 0.62 and 0.72 for SYBR Green I and SYTO9, respectively.

### 4.2. Analysis of Variance (ANOVA)

The ANOVA results for the selected model and the estimated coefficients are summarized in Table 4. A *p*-value less than 0.05 shows that the model was significant. The results revealed that the dye concentration (term A) was significant, unlike term B (incubation time), which was insignificant for both dyes, while term C (staining temperature) was significant only for SYTO9 (*p* < 0.05).

**Figure 3:**
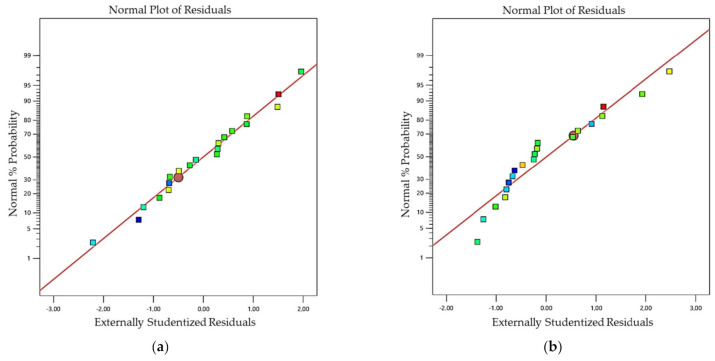

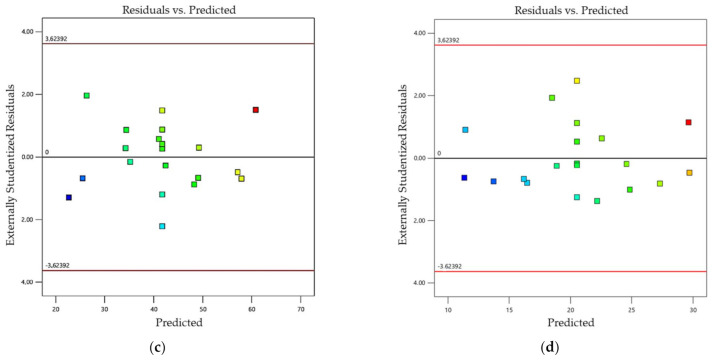
Diagnostic plots of normal plot of residuals for (**a**) SYBR Green I, and (**b**) SYTO9; and residuals vs. predicted for (**c**) SYBR Green I, and (**d**) SYTO9.

The lack-of-fit F- values of 0.45 and 0.64 revealed that the lack-of-fit was not significant, compared to the pure error for both dyes. The diagnostic plots for the normal probability plot of studentized residuals and the studentized results vs. the predicted values are shown in Figure 3. The normal plot of residuals was approximately linear, without significant outliers (Figure 3a,b). The plot of residuals vs. the predicted values (Figure 3c,d), showed a random bounce around the zero line, with an almost horizontal band and no significant outliers. Therefore, it was concluded that, for both dyes, the proposed models were a very good description of the experimental data.

Based on the significance tests (Table 4), it was observed that the dye concentration significantly influenced the viability of the *C. zofingiensis* cells stained with the fluorescent dyes, studied here. These results also revealed that cell damage increased when the dye concentration was increased. This may have been a consequence of the altered physicochemical properties of the membranes, which may have improved their breakthrough, as was observed by Deng et al. [15], who studied the impact of SYTO9 and Propidium Iodide on *Brevibacillus brevis*. A similar effect was shown by Manini and Danovaro [61] and by Shi et al. [62], who observed changes in the percentage of the dead bacteria cells detected at different concentrations of Propidium Iodide. These findings highlight the importance of taking into consideration the concentration to be used for microalgae cell staining, especially when using high concentrations that could severely affect the experiment’s outcome and the interpretation of the results, particularly in the case of viability studies.

The staining temperature was revealed not to be significant when using SYBR Green I in this study, unlike the results found by Hammes et al. [19] and Prest et al. [63], who demonstrated that the staining temperature had a considerable influence on the bacteria cell concentration when using SYBR Green I. However, in our results, the staining temperature has been shown to be significant when using SYTO9 dye. This result highlights the importance of defining specific staining temperatures when using dyes for microalgae cell staining. It also indicates that this is an important parameter, which must be presented by the authors of cell cycle studies, since it has been demonstrated that the temperature influences the staining reaction and its improvement [26].

Although time did not have a significant effect on either dye in this study, Nescerecka et al. [26] showed that it was the most significant factor affecting intact bacteria cell concentration in their study on bacteria viability, using SYBR Green I and Propidium Iodide.

### 4.3. Protocol Optimization and Validation

Once a satisfactory optimization model has been selected, its predictiveness should be verified to ensure that the model is adequate for system approximation [51]. To validate the predictive power of the RSM models in this study, experiments were performed using the predicted optimum conditions of the dye concentration, incubation time and staining temperature for each dye, applying the damaging effect as a key condition to select the optimal level for the factors studied.

The predicted and measured parameters under optimized variable conditions are shown in Table 5. The optimized process parameter configurations suggested by the RSM model, are a dye concentration of 0.5 X and 0.5 µM for SYBR Green I and SYTO9, respectively, with 5 minutes’ incubation time at 25 °C for both dyes.

The measured percentage of damaged cells and the staining efficiency of SYTO9 were close to the predicted values. However, for SYBR Green I, the measured percentage of damaged cells was considerably lower than the predicted one (10% less) and there was a slight difference in the staining efficiency.

**Figure 4:**
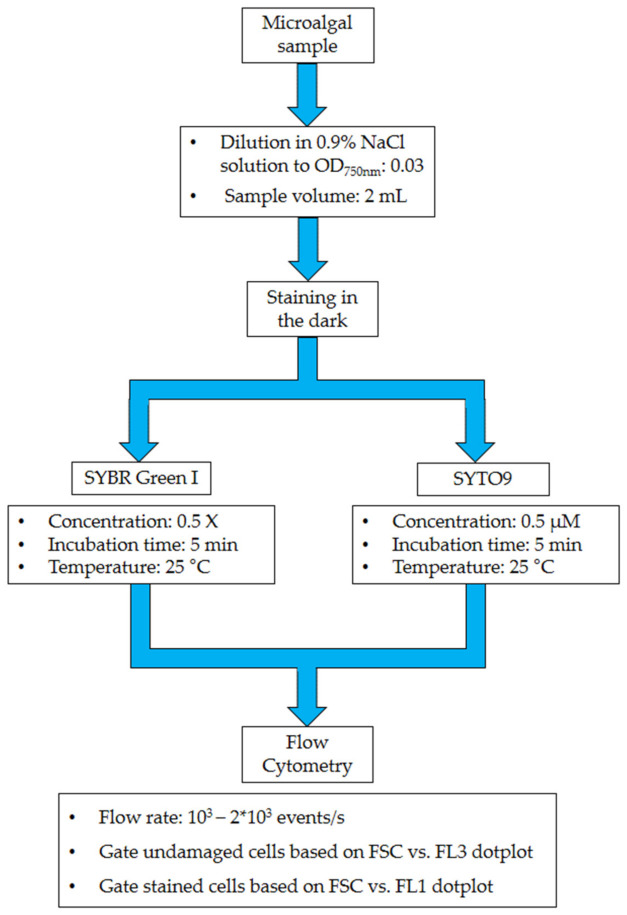
Schematic representation of the validated protocols.

These validated protocols (Figure 4) were also tested on *Chlorella vulgaris,* under the same conditions as above, obtaining a percentage of damaged cells of 8.83% and 8.23%, with a staining efficiency of 99.87% and 99.92%, for SYBR Green I and SYTO9, respectively. These observations indicate that microalgae species could react differently when they are subjected to the fluorescent dyes studied here. In addition, the results from the model validation indicate its appropriateness and precision as a staining routine for both *C. zofingiensis* and *C. vulgaris*. However, regarding the above-mentioned results and considering the literature, adaptations to these protocols are needed to make them applicable to other microalgae species, apart from *Chlorella*.

## 5. Conclusions

The goal of this research was to develop a routine for *Chromochloris zofingiensis* DNA staining, based on flow cytometry measurements. Our approach, using response surface methodology (RSM), allowed us to study and observe how SYBR Green I and SYTO9 affect microalgae cells as a function of three independent factors.

The results showed that the dye concentration is the most important factor to consider, especially for viability studies, alongside the temperature, which can vary depending on the dye used and the species stained. In addition, we demonstrated that the validated protocols can be extended and applied to *Chlorella vulgaris*, but that adaptations must be made so that they can be applied to other microalgae species.

The routine developed in this paper can be used to estimate the nuclear DNA content in cell cycle studies, by means of flow cytometry.

## Figures and Tables

**Table 1:** Coded and actual levels of the independent variables for the central composite design experiments.

Independent Variables	Symbols	Unit	−1 Level	0 Level	+1 Level
Dye Concentration	A	X *–µM **	1.21 *–0.6 **	2.25 *–0.75 **	3.3 *–0.9 **
Incubation Time	B	min	07	10	13
Staining Temperature	C	°C	22	25	28

* SYBR Green I/** SYTO 9.

**Table 2:** Central composite design and results for the staining efficiency and damaging effect of both dyes studied.

Run	Dye Concentration [X *–µM **]		Time [min]		Temperature [°C]		Staining Efficiency [%]		Damaged Cells [%]	
	*	**	*	**	*	**	*	**	*	**
01	2.25	0.6	10	7	20	22	99.83	99.76	51.29	18.19
02	3.3	0.75	13	10	28	25	99.79	99.71	42.37	16.74
03	1.21	0.75	7	10	28	20	99.84	99.76	38.32	22.1
04	2.25	0.75	10	10	30	25	99.81	99.87	36.24	23.94
05	1.21	0.75	13	10	22	25	99.87	99.85	40.21	27.12
06	2.25	0.9	10	7	25	28	99.65	99.79	26.74	24.07
07	4	0.9	10	7	25	22	99.82	99.81	70.52	28.41
08	3.3	1	7	10	28	25	99.8	99.86	44.53	32.71
09	2.25	0.75	10	15	25	25	99.76	99.85	52.57	23.33
10	2.25	0.9	15	13	25	28	99.8	99.77	44.99	18.57
11	1.21	0.9	7	13	22	22	99.79	99.8	34.21	25.08
12	1.21	0.6	13	7	28	28	99.74	99.72	20.92	11.7
13	2.25	0.75	5	5	25	25	99.78	99.81	40.55	24.3
14	2.25	0.5	10	10	25	25	99.72	99.78	32.79	13.89
15	3.3	0.6	13	13	22	28	99.85	99.71	53.79	9.6
16	2.25	0.75	10	10	25	25	99.83	99.82	44.99	19.99
17	3.3	0.75	7	10	22	30	99.84	99.75	53.25	14.35
18	2.25	0.75	10	10	25	25	99.84	99.83	48.41	22.19
19	2.25	0.6	10	13	25	22	99.79	99.75	43.85	14.31
20	0.5	0.75	10	10	25	25	99.67	99.82	14.16	19.8

* SYBR Green I/** SYTO 9.

**Table 3:** Summarized statistics for model fit and results of lack-of-fit test.

Damaged Cells						
Source	SYBR Green I					
	SS	DF	MS	F-Value	*p*-Value	Adjusted R^2^
Linear *	480.53	11	43.68	0.4534	0.8726	0.618
2FI	380.35	8	47.54	0.4935	0.8212	0.579
Quadratic	378.11	5	75.62	0.7849	0.6015	0.454
Cubic	13.99	1	13.99	0.1453	0.7188	0.476
Pure error	481.7	5	96.34			
	**SYTO9**					
Linear *	92.49	11	8.41	0.6395	0.7511	0.719
2FI	91.44	8	11.43	0.8693	0.5914	0.656
Quadratic	40.7	5	8.14	0.619	0.6942	0.6977
Cubic	29.67	1	29.67	2.26	0.1934	0.548
Pure error	65.74	5	13.15			

* Suggested model. SS—sum of squares, DF—degree of freedom, and MS—mean squares.

**Table 4:** ANOVA, *p*-values and F- values of the parameters up to a cell damaging response.

Source	SYBR Green I		SYTO9	
	F-Value	*p*-Value	F-Value	*p*-Value
Model	11.26	0.0003 *	17.21	<0.0001 *
A—Dye Concentration	29.28	<0.0001 *	40.53	<0.0001 *
B—Incubation Time	0.0375	0.8488	2	0.1763
C—Staining Temperature	4.48	0.0504	9.11	0.0082 *
Lack of Fit	0.4534	0.8726	0.6395	0.7511

* represents *p* < 0.05.

**Table 5:** Predicted and experimentally determined number of responses under optimized variable conditions (n = 4).

Staining Dye	Responses							
	Optimum Conditions			Desirability	Damaged Cells [%]		Staining Efficiency [%]	
	Dye Concentration	Incubation Time	Staining Temperature		Predicted	Actual	Predicted	Actual
SYBR Green I	0.5 X	5 min	25 °C	1	23.32	13.75	99.77	99.60
SYTO9	0.5 µM	5 min	25 °C	0.93	13.43	12.86	99.74	99.80

## Data Availability

The data presented in this study are openly available in [Mendeley Data] at [https://data.mendeley.com/datasets/frtr342ghd/draft?a=db2c5077-e8b3-4f1a-a474-aff0fc277f95].
